# Laparoscopic pancreatic duct exploration, electrohydraulic shock wave lithotripsy combined with internal drainage for pancreatic duct stones: A case report

**DOI:** 10.1016/j.ijscr.2023.108190

**Published:** 2023-04-13

**Authors:** Vu Van Quang, Le Trung Hieu, Le Van Loi, Dang Kim Khue, Truong Ngoc Tra My, Le Van Thanh

**Affiliations:** a Hepatobiliary and Pancreatic Surgery Department, 108 Military Central Hospital, Hanoi 100000, Viet Nam; b College of Health Sciences, VinUniversity, Hanoi 100000, Viet Nam

**Keywords:** Chronic pancreatitis, Pancreatic duct stones, Laparoscopic surgery

## Abstract

**Introduction:**

Chronic pancreatitis is characterized by irreversible structural damage, including fibrosis and compression of the pancreatic ducts, often leading to stones forming in the pancreatic duct and parenchyma. Surgery is indicated when severe obstruction with chronic pain is presented and conventionally drained by pancreatojejunostomy.

**Case report:**

A 56-year-old female patient with epigastric pain for many years. Computed tomography revealed an atrophic pancreas with a dilated pancreatic duct (18 mm) obstructed by a stone sized 1.3 cm. The patient underwent laparoscopic pancreatic duct exploration, used electrohydraulic lithotripsy for pancreatic duct stones, and then placed pancreaticoduodenal internal drainage with primary closure of the pancreatic duct. The operative time was 185 min, and the total blood loss was around 50 ml without intraoperative complication. The patient was discharged from the hospital on postoperative day 5 uneventfully. The epigastric pain symptoms dramatically decreased in the follow-up visit after one month.

**Clinical discussion:**

We combined several minimally invasive techniques to treat a chronic pancreatitis patient with a stone forming in the main duct in this patient. We used lithotripsy and internal drainage without the need for anastomosis. To our knowledge, this is the first report on this technique in literature. We found this technique is safe and applicable in selected patients to treat pancreatic stones with the dilated pancreatic duct.

**Conclusions:**

In this case, we demonstrate a novel surgical treatment option for chronic pancreatitis with a simple and effective technique to manage pancreatic stones in chronic pancreatitis patients.

## Highlights

• Chronic Pancreatitis is a chronic condition with irreversible structural damage.
• Pancreatic stones were formed in most chronic pancreatitis cases, resulted in pain caused by obstructed pancreatic duct.
• We proposed a minimally invasive technique to manage pancreatic duct stone with primary closure of the pancreatic duct.

## 1 Introduction

Chronic pancreatitis is a condition characterized by irreversible structural damage, including fibrosis and compression of the pancreatic ducts. It leads to exocrine and endocrine dysfunction, manifesting as diabetes and chronic pain. 50 % of patients with chronic pancreatitis have stones forming intra pancreatic duct. Alcohol consumption and smoking are well-documented risk factors for this condition [1]. Research has shown that 40–75 % of patients with chronic pancreatitis do not respond to medical therapy and endoscopic intervention, necessitating the consideration of surgery, often due to persistent abdominal pain. The surgical objective is to achieve effective and lasting pain relief, minimize early and late complications, and maintain the structural and functional integrity of the pancreas [1], [2].

Pancreatic duct exploration and drain with pancreatojejunostomy are conventional operations for managing pancreatic duct stones. Currently, laparoscopic drainage is an emerging technique for treating chronic pancreatitis, offering advantages such as smaller incisions resulting in better cosmetic results, reduced postoperative pain, quicker recovery, and shorter hospital stays. However, laparoscopic surgery has limitations in removing large stones partially embedded in the pancreatic tissue, which increases the risk of bleeding. Additionally, the anastomosis modifies normal gastrointestinal anatomy and physiology. To address these limitations, we present our initial experience with a laparoscopic technique involving pancreatic duct exploration (LPDE), electrohydraulic lithotripsy, and internal drainage without pancreatic-enteric anastomosis for pancreatic duct stones treatment. This case presentation follows SCARE guideline [3].

## 2 Case report

**Fig. 1:**
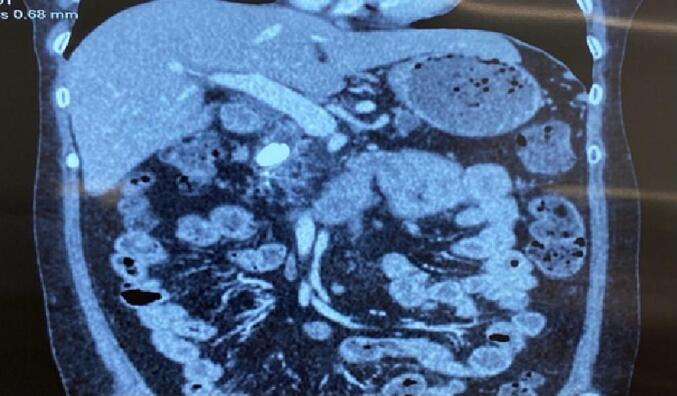
CT scan imaging: Pancreatic headstone.

**Fig. 2:**
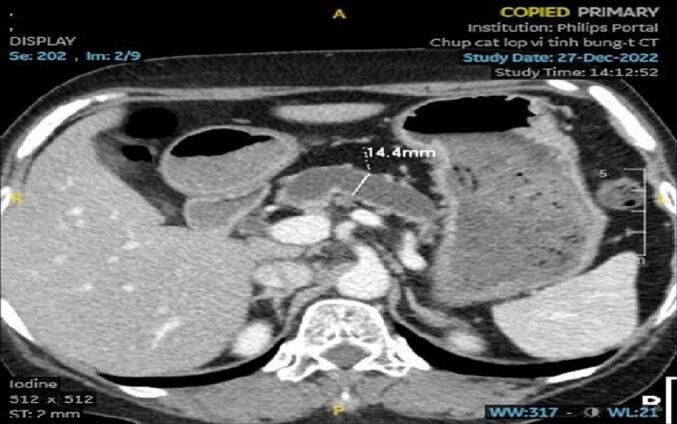
CT scan imaging: Dilated pancreatic duct.

A 56-year-old female patient with a history of hypertension presents with a three-year history of epigastric abdominal pain, which has increased in intensity over the past month and now radiates to the back. She has no history of alcohol consumption, diabetes, or other remarkable medical history. All her blood tests were within normal limit. The computed tomography scan of the upper abdomen revealed pancreatic atrophy and a dilated pancreatic duct (18 mm) with the presence of large stones (1.0 cm × 0.6 cm) in the pancreatic head (Fig. 1, Fig. 2). No malignancy or biliary dilation was detected. The preoperative diagnosis is chronic pancreatitis with dilated duct obstructed by a large stone.

A laparoscopic exam of the pancreatic duct was performed, followed by applying electrohydraulic lithotripsy. The duct was then emptied into the duodenum using an 8 Fr catheter, and the opening was closed with PDS 4/0 sutures without creating pancreatojejunal anastomosis.

**Fig. 3:**
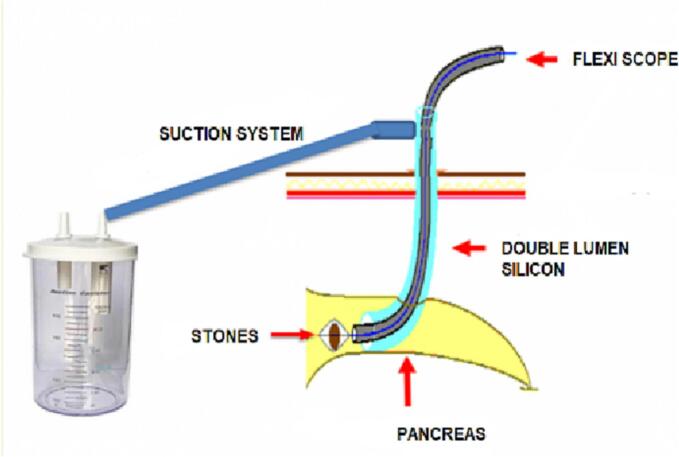
Lithotripsy system.

**Fig. 4:**
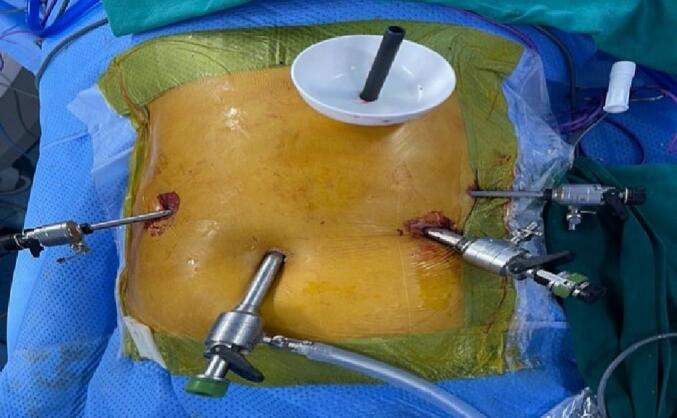
Trocars positions and lithotripsy system.

Before the procedure, the patient was administered prophylactic antibiotics and endotracheal anesthesia. The patient was positioned in a reverse Trendelenburg position with legs apart and tilted 30 degrees to the right. Five trocars were utilized, including two 10 mm trocars, two 5 mm trocars, and one 10 mm trocar located directly at the opening of the pancreatic duct, to ensure the placement of a connecting tube from the duct to the skin, preventing the entry of stones or fluid into the abdomen (Fig. 3, Fig. 4).

**Fig. 5:**
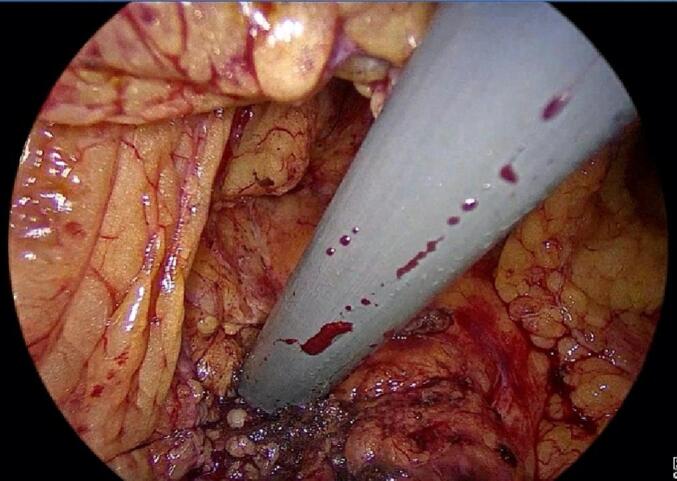
Insertion of the catheter into the pancreas.

We performed a laparoscopic evaluation of the abdominal cavity and opened the great omentum to access the anterior surface of the head and body of the pancreas. This was achieved by elevating the stomach and lowering the transverse colon. Using a unipolar electric knife, we incised the pancreatic duct to a diameter of 8 mm, which was large enough for inserting the percutaneous catheter at the head and body of the pancreas (Fig. 5). The flexi endoscope was then inserted into the pancreatic duct to assess and evaluate its condition and the location of pancreatic stones.

**Figs. 6, 7:**
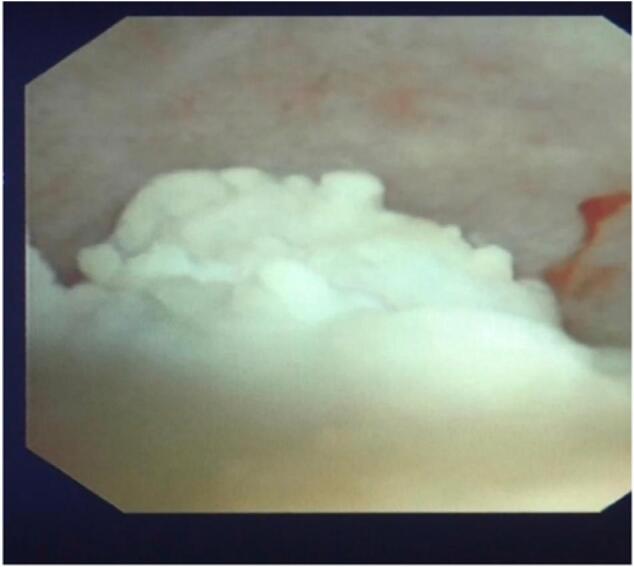

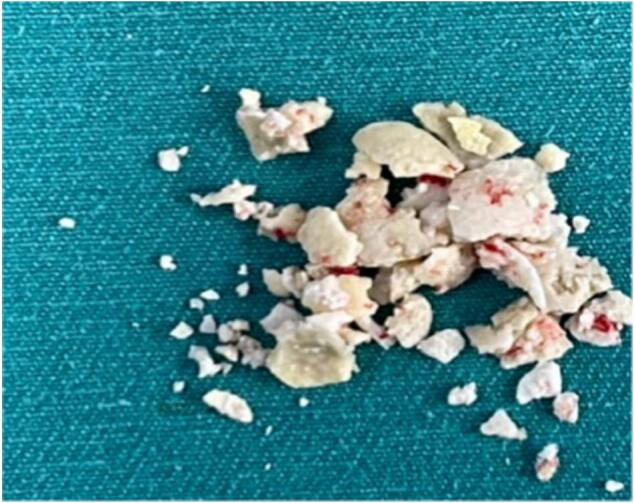
Endoscopic images of pancreatic stones and stones after surgery.

**Fig. 8:**
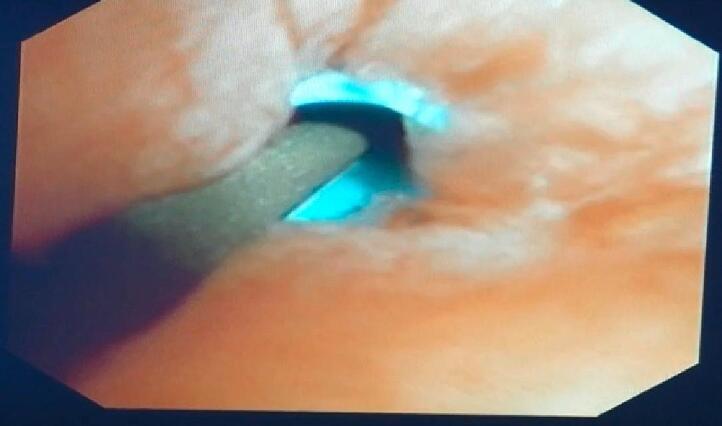
Insertion of trans pancreatic duct catheter to the duodenum.

We performed hydraulic wave pancreatic lithotripsy to break up the stones into smaller pieces and remove them by stone extraction baskets, suction, and irrigation of the pancreatic duct (Figs. 6, 7). After removing the stones, we ensured that no residual stone debris or bleeding was present in the duct. Then, we inserted an 8 Fr drainage into the main pancreatic duct to drain its contents (Fig. 8) and then closed the opening with PDS 4-0 sutures without performing pancreatojejunal anastomosis.

**Fig. 9:**
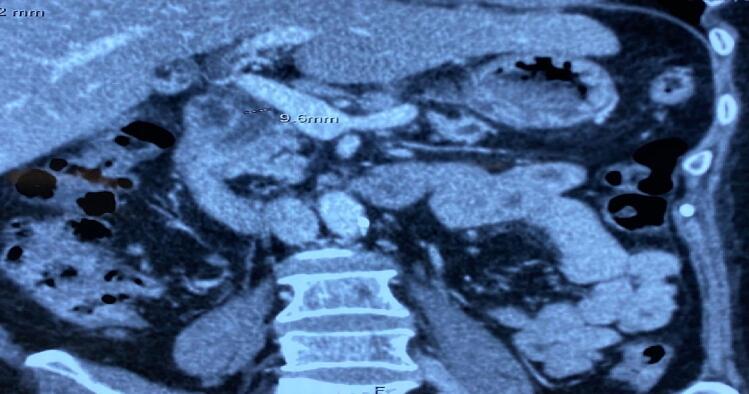
CT scan 3 months after surgery shows no stones left.

The surgical procedure was finished without placing abdominal drainage and lasted for 185 min, with a total blood loss of 50 m. We administered routine laboratory tests on postoperative day 1 and 3, without any sign of acute pancreatitis. The patient was discharged on the fifth day after the procedure uneventfully. After three months, the patient reported a significant decrease in pain, and a follow-up abdominal CT scan showed no presence of stones (Fig. 9). We removed the catheter by endoscopy.

## 3 Discussion

Chronic pancreatitis is a debilitating condition with multiple etiologies characterized by parenchymal destruction and structural alteration of the pancreatic duct. Stones in the pancreatic duct are common in 90 % of patients suffering from chronic pancreatitis. These stones obstruct the duct and cause elevated pressure and ischemia, leading to abdominal pain. Pain, either continuous or episodic, is the primary and most severe symptom of this disease, severely impacting the patient's quality of life.

The surgical indications should consider the affected pancreatic tissue's location and the pancreatic duct's dilation. Drainage procedures and pancreatectomy are the two main surgical approaches to improve pancreatic duct drainage in chronic pancreatitis patients. Both options aim to reduce pain and preserve as much pancreatic tissue as possible while ensuring safety [4], [5], [6]. The modified Partington-Rochelle lateral pancreatojejunostomy is widely recognized as a safe and effective surgical drainage method, as it maximizes the preservation of pancreatic tissue. However, it does not address the underlying cause of the disease, as the inflammatory mass remains in the head of the pancreas, and the procedure requires pancreatojejunostomy anastomosis, altering the normal anatomy of the gastrointestinal tract. The appropriate indication for drainage surgery is in patients with a solitary pancreatic duct (dilation > 7 mm) without an inflammatory mass in the head of the pancreas and has shown long-term pain relief of 60–70 % in this patient population. Conversely, some authors reported that recurrent pancreatitis might occur in the head of the pancreas in about 30 % of patients who do not undergo pancreatojejunostomy, as the ducts of Wirsung or Santorini and their branches remain undrained [7].

To our knowledge, this is the first report on a laparoscopic approach to treating pancreatic duct stones through an opening of the duct, followed by lithotripsy and pancreaticoduodenal drainage via an 8Fr catheter in the medical literature. This case report presents the successful implementation of this novel technique. The approach offers several benefits, including clear visualization of the pancreatic duct, precise stone location identification, reduced tissue damage during stone removal using hydraulic lithotripsy and stone extraction baskets, and decreased risk associated with pancreatoenteric anastomosis. Additionally, the direct drainage of the pancreatic duct into the duodenum via a percutaneously placed silicone tube with a diameter of 10 mm results in minimal blood loss and accelerated recovery. It is important to note that this technique only applies to cases where the diameter of the pancreatic duct is >8 mm to accommodate the diameter of the trocar required for placement of the endoscopic lithotripsy tunnel. The silicone tube is fitted with an innovative dual-channel connector to pass the endoscope and install a suction tube for fluid and stone removal. The horizontal design of the suction tube prevents the collapse of the pancreas during the procedure. The one-way closed system used in the pancreatic lithotripsy process provides a more convenient and efficient operation and reduces the risk of spillage and infection. Internal stent placement with primary closure of the pancreatic duct serves to maintain the normal anatomy of the gastrointestinal tract and decrease pressure within the pancreatic duct.

Liu et al. [8] report that laparoscopic surgery is indicated for cases with a maximum of three stones and a stone diameter of 10 mm or less in the head or body of the pancreas. If the stone is in the main pancreatic duct and is small, removal is more likely to be successful [9], [10]. Stones scattered in the pancreatic duct or located in the lateral branch ducts is challenging in removal [11], [12].

Other minimally invasive approaches for pancreatic stones are endoscopic retrograde cholangiopancreatography (ERCP) lithotripsy and extracorporeal shockwave lithotripsy (ESWL). The ERCP lithotripsy is challenging and needs experienced endoscopist with potential severe complications. In the other hand, the use of ESWL as a therapeutic strategy for the management of pancreatic duct stones has extensive range of indications, especially endoscopic approach in conjunction with ESWL offers a promising solution to remove stones located in both the main and accessory pancreatic ducts [13], [14], [15], [16]. The guidelines established by the European Society of Gastroenterology firmly position ESWL as the preferred treatment option for patients diagnosed with pancreatic duct stones measuring ≥5 mm in the main duct [17], and is contraindicated in patients with pregnancy, at risk of bleeding, or have pacemakers, defibrillators, or abdominal aortic aneurysms [18]. However, several studies have been conducted to compare the clinical outcomes of endoscopic and surgical treatments for Pancreatic Duct Stones (PDS). The results showed that surgery was more effective in achieving complete or partial pain relief than endoscopic therapy [19], [20].

This case report provides evidence of the feasibility of laparoscopic pancreatic duct incision, lithotripsy, and pancreaticoduodenal drainage, followed by primary closure of the pancreatic duct opening to treat chronic pancreatitis. Via the opening of the pancreatic duct, we can easily control the bleeding and remove stone fractions. We indicate this procedure for cases with the stones located solely in the main pancreatic duct, and the duct dilated >8 mm, and there is no enlargement of the pancreatic head tissues or suspected lesions. The goal of surgery in PDS treatment is to remove the obstructing pancreatic duct stones while preserving pancreatic function through decompression of the duct. Our new technique aims to completely dissolve the stones and place internal drainage, without the need for pancreatoenteric anastomosis and preserving the anatomical structure of the gastrointestinal tract. However, it should be noted that this study is limited to our first patient and follow-up period and that further investigation, including larger sample sizes and more extended follow-up periods, is needed to validate these findings and compare the efficacy of this technique with other available approaches.

## 4 Conclusion

In this case, we demonstrate a novel surgical treatment option for chronic pancreatitis. It is safe and effective management in cases with dilated pancreatic ducts that can be achieved using laparoscopic pancreatectomy, electrohydraulic lithotripsy, and internal drainage.

## Informed consent

Written informed consent was obtained from the patient for publication of this case report and accompanying images. A copy of the written consent is available for review by the Editor-in-Chief of this journal on request.

## Provenance and peer review

Not commissioned, externally peer-reviewed.

## Ethical approval

This study was approved by Institutional Ethical Committee.

## Funding

The study did not receive external funding.

## Guarantor

Le Van Thanh.

## Research registration number

8844

## CRediT authorship contribution statement

**Van Quang Vu:** Main surgeon, Methodology, Writing - original draft.
**Trung Hieu Le:** Methodology, Writing - original draft.
**Van Loi Le:** Surgeon, Assisting the operation.
**Kim Khue Dang:** Advising, Editing and Submitting the manuscript.
**Ngoc Tra My Truong:** Writing manuscript, Editing English.
**Van Thanh Le:** Oversee the project, Final approval of the manuscript.

## Conflict of interest

We have no conflicts of interest to disclose.
